# Reimagining zoonotic malaria control in communities exposed to *Plasmodium knowlesi* infection

**DOI:** 10.1186/s40101-022-00288-y

**Published:** 2022-04-12

**Authors:** Nurul Athirah Naserrudin, April Monroe, Richard Culleton, Rozita Hod, Muhammad Saffree Jeffree, Kamruddin Ahmed, Mohd Rohaizat Hassan

**Affiliations:** 1 Department of Community Health, Faculty of Medicine, Universiti Kebangsaan Malaysia, Cheras, Kuala Lumpur, Malaysia; 2 Borneo Medical and Health Research Centre, Faculty of Medicine and Health Sciences, Universiti Malaysia, Kota Kinabalu, Sabah Malaysia; 3 Sabah State Health Department, Ministry of Health, Kota Kinabalu, Malaysia; 4 Johns Hopkins Center for Communication Programs, Johns Hopkins Bloomberg School of Public Health, Baltimore, USA; 5 Department of Molecular Parasitology, Proteo-Science Centre, Ehime University, Matsuyama, Japan; 6 Department of Public Health, Universiti Malaysia Sabah, Kota Kinabalu, Malaysia

**Keywords:** *Plasmodium knowlesi*, Zoonotic malaria, Reimagining, Human behavior, Community engagement, Community participation, Primary prevention, Malaria control

## Abstract

*Plasmodium knowlesi* malaria infection in humans has been reported throughout southeast Asia. The communities at risk are those living in areas where *Macaque* monkeys and *Anopheles* mosquito are present. Zoonotic malaria control is challenging due to the presence of the reservoir host and the possibility of human-vector-human transmission. Current control measures, including insecticide-treated nets (ITNs) and indoor residual spraying (IRS), are insufficient to address this threat due to gaps in protection associated with outdoor and early evening vector biting and social and economic activities, such as agricultural and forest work. Understanding the challenges faced by affected communities in preventing mosquito bites is important for reducing disease transmission. This opinion paper discusses opportunities to improve *P. knowlesi* malaria control through understanding the challenges faced by communities at risk and increasing community engagement and ownership of control measures. The paper highlights this issue by describing how the concept of reimagining malaria can be adapted to zoonotic malaria control measures including identifying current gaps in vector control, understanding interactions between environmental, economic, and human behavioral factors, and increasing community participation in and ownership of control measures.

## Background

Malaria is a mosquito-borne-infectious disease, which has a global distribution and significant health burden [1]. Malaria is caused by a parasite from the genus *Plasmodium* and transmitted by the *Anopheles* mosquitoes [1]. Globally, the predominant parasites and species responsible for human malaria include *P. falciparum*, *P. vivax*, *P. malariae*, and P*. ovale* [1]. The first naturally acquired human infection with *P. knowlesi* malaria was detected in 1965 in a United States (US) army surveyor returning from Malaysia [1]. In 2004, a large group of human populations was identified with *P. knowlesi* infection in Kapit, Sarawak, Malaysia [1]. This *Plasmodium* species , which was rarely found in humans, has since been called the fifth species of human malaria [2]. Ever since then, an exponential increase in confirmed *P. knowlesi* malaria cases has been identified among the population in the southeast Asian region, such as Malaysia, Indonesia, the Philippines, Vietnam, Cambodia, and Thailand [2–4]. This event signals a potential public health threat associated with zoonotic malaria [2, 4].

The efforts to eliminate human malaria have been established globally. Meanwhile, the World Health Organization (WHO) has recognized the regional importance of *P. knowlesi* malaria affecting the southeast Asian countries [4]. The Evidence Review Group (ERG) meeting on *P. knowlesi* malaria has reviewed evidence from studies and recommended strategies for the prevention and control of this zoonotic infection [4]. The threat and challenges to control of this malaria parasite species have emerged as an important cause of indigenous malaria in some southeast Asian regions [4]. The WHO included *P. knowlesi* malaria statistics and the importance of its control in the World Malaria Report in 2020 [1].

*P. knowlesi* malaria is a nonhuman parasite found in the natural host, which comprises of the *Macaque* monkeys and *Prebystys* species (banded leaf monkeys) [4]. The spillover of the infection in humans has caused dynamic changes in disease transmission, as evidenced by asymptomatic infection in humans [4]. To date, there are no reports of natural human-vector-human transmission of *P. knowlesi* infection, and current studies reflect that this malaria remains a zoonotic infection [4, 5]. There is a possibility of human-to-human transmission in high-risk areas; however, more research focusing on genetic study is needed to prove or disprove the hypothesis [4]. The boundaries of the infection are due to the natural distribution of the *Macaques* monkey and *Anopheles* vectors [3, 4]. Meanwhile, there are no reports of *P. knowlesi* malaria infection outbreaks in humans, in areas lacking the natural reservoir of the disease [4]. The ERG and the Malaria Policy Advisory Group (MPAG) recommended future research to explore in-depth prospective epidemiological and laboratory genomic studies, to find evidence of the possibility of human-vector-human transmission [5]. The emerging threat of this zoonotic infection may be altered by the parasite’s ability to adapt over time, thus threatening malaria elimination goals in some regions [4]. Further research on *P. knowlesi* malaria should explore and identify effective interventions that suit local conditions. The multicollaborative efforts are urgently needed to limit malaria transmission in the current concentrated areas and other susceptible to *P. knowlesi* malaria transmission.

The state of Sabah, located on the Malaysian Borneo Island, reported the highest incidence of human *P. knowlesi* malaria cases despite Malaysia’s progress in eliminating indigenous human malaria [5]. In Sabah, a total of 4131 cases were detected with incidence rate of 0.13 per 1000 population in 2018, which is a significant increase compared to 376 cases reported in 2008 [6]. The increase in *P. knowlesi* malaria cases in this region has been attributed to several factors, such as improved diagnostic screening, increased awareness of *P. knowlesi* infection in the population, loss of relative immunity due to lower rates of human malaria exposure, and changes in land use causing zoonotic spillover of infections from *Macaque* monkeys to humans [4]. The anthropogenic activities and environmental changes in Malaysian Borneo Island, such as rapid deforestation and agricultural expansion (e.g., rubber, coconut, and palm oil plantations), contributed to the zoonotic spillover of this infection to human [3, 4, 6, 7]. This upward trend of *P. knowlesi* cases in human population is forecasted to increase in the coming years [8].

Before 2008, the misdiagnosis of *P. knowlesi* cases as *P. malariae* infection was attributed to the use of blood smears on light microscopy (LM), which is a less sensitive and specific diagnostic screening method [2]. Nevertheless, the challenge in differentiating these species has been remarkably reduced by using the polymerase chain reaction (PCR) [2]. Although most malaria cases were uncomplicated with a wide range of symptoms such as fever, myalgia, arthralgia, and headache, these nonspecific symptoms are often insufficient to provide an accurate diagnosis [4, 6]. An estimated > 10% of patients developed severe malaria in Sarawak, Malaysia [4]. However, the are no cerebral malaria complications in *P. knowlesi* malaria as compared to the life-threatening *P. falciparum* cases [1].

Ecological changes have driven behavioral changes in the vectors and reservoir and have caused spillover infections to humans [9]. Sociodemographic factors play a significant role in disease exposure, with adult males, agricultural workers, and farmers experiencing the highest risk of exposure [4, 10]. Moreover, asymptomatic *P. knowlesi* cases have been detected across all ages and sociodemographic groups, including children and women [11, 12]. The risk of exposure in the vulnerable population requires urgent attention to identify and measure key human-vector interactions and human activities. The detailed information on nighttime activities of different groups at risk of malaria exposure could serve as a foundation to inform context-specific research towards a more locally effective intervention [13]. The dynamic of the parasite’s rapid life cycle during the erythrocytic stage is critical in infected individuals. Specifically, *P. knowlesi* cause high parasitemia within 24 h during the erythrocytic stage, which is associated with severe malaria. The parasetemia level and age of cases are independently associated with severe *P. knowlesi* disease [4]. The parasetemia level and age of cases are independently associated with severe *P. knowlesi* disease [4]. All *P. knowlesi* cases with > 15,000 parasites/ul require urgent treatment and close monitoring [4]. While most human cases are uncomplicated, fatalities have been reported in severe cases in young individuals as low as 32 years old [4]. The case fatality rate was 1.70/1000 (95% confidence interval [CI], 1.66–1.75) in Sabah, Malaysia [14], with females and individuals aged 45 years and above account for 50% of the mortality cases and at a higher risk (*OR* 4.7 [95% *CI* 1.8–12.5) upon adjusting for gender [14]. These findings highlight the need for alternative methods to facilitate more sensitive and specific disease detection, as well as intervention tailored to specific age groups and gender.

The perimeter of *P. knowlesi* malaria exposure is bounded by human activities in and near the forest harboring the natural simian reservoir and *Anopheles* mosquitoes. The epidemiological triad of *P. knowlesi* needs a strong interaction between these elements. Outbreaks of human malaria cases have been reported in urban areas [15]. Furthermore, malaria outbreaks have occurred due to climatic changes in metropolitan and big cities like Moscow in Russia and New York in the USA [15]. Climatic factors such as temperature, rainfall, humidity, and wind influence the life cycle of mosquitoes while indirectly contributing to the multiplication of the parasite [16]. While there are research gaps on climate change concerning *P. knowlesi* outbreaks, previous evidence has identified climatic factors as contributing factors to autochthonous transmission [15]. Additionally, the effects of global warming have heightened the risk of vector-borne diseases including malaria in recent decades [16]. Thus, it is crucial to determine the effect of deforestation on future *P. knowlesi* disease as the exposure is strongly altered by anthropogenic activities. A critical investigation and evaluation to identify the effect of climate change as a triggering factor for the increasing cases of *P. knowlesi* malaria in humans are required to improve antimalarial preparedness against possible outbreaks in high-risk malaria regions.

Despite the high incidence of *P. knowlesi* infection, the number of cases might be underreported [6]. This zoonotic malaria is a complex disease, and its dynamic epidemiology contributes to the challenge of effectively controlling the disease [5]. In addition to deforestation and the possibility of climate change to future *P. knowlesi* outbreaks, the presence of the *Macaque* monkey as the natural host threatens the malaria elimination strategy. The conducive environment for mosquito breeding favors the resurgence of malaria [15]. In vulnerable communities, sound epidemiological and public health strategies in linking human activities with entomological parameters would be more plausible in malaria exposure [13]. The possibility of sustained human-vector-human transmission, without the involvement of primates in the epidemiological chain, requires innovative strategies in research and to combat further transmission [4, 16].

While biomedical, epidemiological, and technological research and solutions are important, success will also depend on a deeper understanding of social, economic, and human behavioral factors [17]. This information can improve health outcomes by ensuring interventions are tailored to the local context [17–19]. Engagement with affected communities in malaria control programs, and a clear understanding of the factors driving or inhibiting malaria-related behaviors, can facilitate effective social and behavior change (SBC) programs, increase the uptake of protective measures, and help ensure the continuity and sustainability of malaria interventions [17, 20].

This opinion paper identifies opportunities to reimagine *P. knowlesi* malaria control through understanding the challenges faced by communities at risk. This includes identifying gaps in vector control measures, exploring the underlying human behavior and social challenges, and integrating community participation and ownership in disease control [17, 20].

### Reimagining zoonotic malaria control

In 2015, *Malaria Journal* initiated an inter- and transdisciplinary dialogue called “reimagining malaria.” It generated constructive criticism and innovative thinking around the current malaria program [21], including the importance of elevating the voices of effected communities and identifying more holistic solutions for sustainable malaria control [21]. While the concept of reimagining malaria has focused largely on human malaria, it is equally important for addressing the threat of zoonotic malaria.

Despite various quantitative studies describing the risk factors contributing to *P. knowlesi* infection [10, 22–24], additional research is needed to more fully understand the challenges faced by vulnerable communities to avoid zoonotic malaria [25–27]. For example, empirical evidence among forest workers in Acheh, Indonesia, described the barriers to preventive measures due to their working environment [25]. Moreover, social challenges like undocumented workers, distance to healthcare facilities, and negative experiences when consulting the healthcare workers contributed to poor health seeking among these communities [25]. Future malaria research should be more holistic and move towards transdisciplinary approaches [21]. This includes integrating incidence and prevalence data with information on environmental, social, cultural, and economic factors. Likewise, solutions must go beyond technical innovation to address social and contextual barriers to participation. The rationale behind the noncompliance to intervention and exposure to malaria could not be explained by quantitative approaches. Hence, an in-depth exploration of populations at risk and their behavior in natural settings is required [17].

Engaging affected communities can provide valuable insights into the effectiveness and feasibility of the ongoing program. In the presence of local perspectives and implementation challenges, this can generate actionable opportunities for improvement in strategies and increase acceptance and accessibility [21]. Modifications to malaria control approaches can be done based on the local context to ensure the sustainability of the malaria program [17, 20]. Like reimagining human malaria, reimagining zoonotic malaria can generate unanticipated insights through research designs using an open-ended process [21] and transdisciplinary approaches to address the cultural, social, environmental, economic, and political determinants of health.

### Identify gaps in vector control measures

Previous research on *P. knowlesi* has included indicators of noncompliance to insecticide-treated nets (ITNs) usage and indoor residual spraying (IRS) as a driver of *P. knowlesi* exposure [10, 28]. However, in recent years, vector studies described *Anopheles* mosquitoes biting outdoors, at earlier hours, just after dark [7, 29, 30]. Moreover, in Sarawak, which is located in Borneo Island, a new group of vectors, the *Umbrosus* group, was found to bite at night and morning hours (0700–1100 h) [31]. Furthermore, vulnerable communities are often involved in farming, agriculture, sleeping outdoors, and hunting [4] — making this type of vector control measure impractical in some settings. Occupational-related challenges can cause barriers to the consistent usage of core vector control interventions and in turn limit their effectiveness [25].

While surveys reported the usage of vector control measures such as ITNs to be high in many contexts [10, 28], ITN use is a nuanced behavior that can vary over the course of the night, across seasons, over time, and among different sociodemographic groups [32, 33]. Fundamental issues on human behavior related to malaria exposure like social activities, gatherings, and nighttime activities require field observation and grounded research to provide details on community lifestyle and patterns of malaria exposure [13, 18, 34–36].

With an increasing number of vector control tools available and in development, it is crucial to better understand human activities that intersect with mosquito biting and determine the time and place when individuals or communities are exposed to mosquito bites to help limit the risk of zoonotic malaria exposure in these vulnerable communities [4, 34]. Complementary vector control tools that are feasible and acceptable to higher-risk individuals and communities should be developed and deployed. Insecticide-treated clothing and hammocks are some of the possible alternative measures that are already available and could be deployed more broadly for personal protection [37]. For communities that keep monkeys as pets and are involved in forest activities where *Macaques* monkeys are present, additional strategies targeting the malaria parasite might also be considered [10, 25, 26]. For example, in Sulawesi, Indonesia, where keeping pet primates is common and habitat destruction, bushmeat hunting, and trapping due to crop raiding increase contact between humans and macaques, complementary measures such as antiparasitic drugs can be used [38].

### Underlying human behavior and social challenges

Current works of literature on risk factors for *P. knowlesi* malaria have identified activities and lifestyles that put humans at risk including forest-related activities like travelling [10, 25, 26, 34, 39, 40], working [23, 25, 34, 40], and sleeping in the forest [25, 34, 39]. However, peri-domestic infection was also detected in households that did not perform forest-related activities. Housing areas surrounded by oil palm, rice paddy fields, and long grass provide a suitable environment that puts individuals at risk *for P. knowlesi* exposure [10]. The relation of peri-domestic environment and human activities highlights the need to better understand these factors and how they relate to malaria risk. Fornace et al., for example, highlighted the importance of exploring human space use in different ecologies in determining the exposure to *P. knowlesi* malaria infection [40]. Likewise, while certain groups are known to be at higher risk of exposure, males and females across all age groups and occupations experience some risk as evidenced by the presence of specific *P. knowlesi* antibodies [11, 24]. While men are often exposed to mosquito bites through forest and agricultural work, it is unclear which activities may be driving transmission in children and women. Activities like socializing, gathering outdoors at night, and even sleeping outdoors are possible risks; however, there has been limited research done on this subpopulation in settings where *P. knowlesi* is present. Further studies are required to better understand the interrelation between human activities and *Anophel*es vector behavior in these contexts [34].

The behavior of mosquitoes that bite outdoors, as early as 1800 h in the evening, and in the morning hours (0700–1100) for the *Umbrosus* group, could be due to vector adaptation to close contact with humans who live and perform activities in/near the forest. Ecological changes due to deforestation, crop planting, and housing were also described to contribute to this phenomenon [30, 40, 41]. Integrating current research on spatial mapping of *P. knowlesi* hotspots [40, 42] and further exploration of the social context and challenges to avoiding mosquito bites in the community is needed. An understanding of individual and community knowledge, attitudes and environmental drivers, and facilitators has provided critical information to improve human malaria strategies [43]. In addition, a recent review has recommended to integrate the understanding of human behavior and its social context to inform future behavior change programs to control zoonotic malaria in these vulnerable communities [34].

### Community participation in disease control

The World Health Organization (WHO) has recommended including community participation as one of the pillars for vector-borne disease control [20] underscoring the importance of collaboration with affected communities and finding solutions that suit the local sociocultural and social environment. The participation of communities in malaria control can not only increase the impact of the program but also help to ensure its sustainability [20]. The Sustainable Development Goals (SDGs) explicitly address the importance of community participation towards a better health outcome by including social determinants of health such as sociocultural and environmental factors [44]. Through the multisectoral approach for vector-borne disease, the WHO highlighted the importance of partnerships across governmental bodies and other sectors and affected communities [20]. However, the social context of vulnerable communities is often overlooked in *P. knowlesi* malaria studies [34].

Community engagement can involve a wide range of activities along a continuum from identifying the social issues to active engagement to community ownership [45]. Currently, passive community participation includes acceptance of spraying of IRS on the home wall and receiving the free ITNs that are provided by healthcare workers. There is an important opportunity to move malaria programs along the continuum toward more meaningful partnerships with affected communities. The presence of asymptomatic *P. knowlesi* malaria underscores the value of actively engaging communities to support prevention, recognition of symptoms, community screening, and health-seeking behavior to prevent outbreaks (or clusters) in these settings.

Initiatives should prioritize community empowerment and strive for active engagement of vulnerable communities in future zoonotic malaria planning. Actively including communities in developing a zoonotic malaria control program through a bottom-up approach can ensure more feasible and accessible malaria control measures. In addition, working closely with marginalized communities in prioritizing malaria interventions that are locally and culturally acceptable promotes greater equity in malaria control strategies.

## Conclusion

**Fig. 1:**
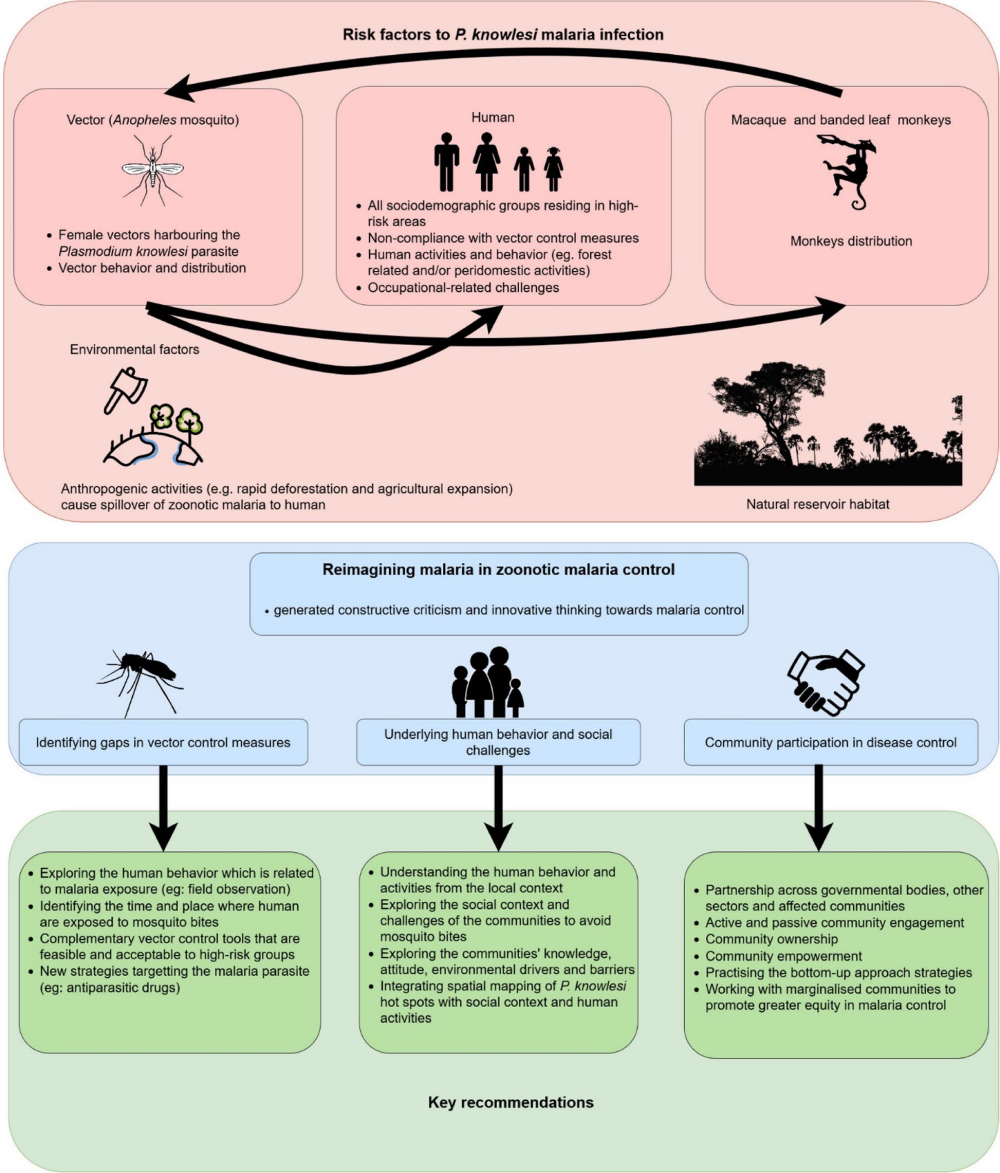
Reimagining zoonotic malaria control in communities exposed to *Plasmodium knowlesi* infection

The concept of reimagining malaria used in human malaria also applies to zoonotic malaria disease control and can point to new methodologies and approaches to increase impact and sustainability (see Fig. 1). Integrating human behavior and social risk factors, vulnerabilities, and environmental challenges can provide information for SBC programs and future *P. knowlesi*-specific malaria control. With the possibilities of human-vector-human transmission, the likelihood of such transmission cannot be ignored. The intersection of social, cultural, and environmental factors that drive zoonotic malaria transmission requires a transdisciplinary approach. Optimization of zoonotic malaria control will depend on strategies that reflect the experiences and priorities of vulnerable communities and an improved understanding of the complex factors shaping patterns of exposure and prevention.

## Data Availability

Data sharing is not applicable to this article as no datasets were generated or analyzed during the current study.

## Abbreviations

US: United States
ERG: Evidence Review Group
MPAG: Malaria Policy Advisory Group
LM: Light microscopy
ITNs: Insecticide-treated nets
IRS: Indoor residual spraying
SBC: Social and behavior change
WHO: World Health Organization
SDGs: Sustainable Development Goals
